# A novel variation of the recurrent laryngeal nerve

**DOI:** 10.1186/s12893-017-0263-5

**Published:** 2017-06-02

**Authors:** Gaosong Wu, Kun Wang

**Affiliations:** 1 Department of Thyroid and Breast Surgery, Zhongnan Hospital of Wuhan University, 169 DongHu Road, Wuhan, People’s Republic of China; 2 Department of Thyroid and Breast Surgery, Tongji Hospital of Tongji Medical College of Huazhong University of Science and Technology, 1095 Jiefang Avenue, Wuhan, People’s Republic of China

**Keywords:** Recurrent laryngeal nerves, Variation, Thyroid surgery, Vocal cord paralysis

## Abstract

**Background:**

Injury to the recurrent laryngeal nerve is one of the most severe complications of thyroid surgery. Several anatomic variations of the nerve increase the likelihood of iatrogenic damage.

**Case presentation:**

A 50-year-old woman was presented to our department with a nodule in the right thyroid lobe, and she reported no voice changes. She had no history of surgery or radiation to the head or neck. Fine-needle aspiration was recorded as papillary thyroid carcinoma. The preoperative laryngoscopy revealed left vocal cord paralysis. Right thyroid lobectomy was performed. A scarce course of the left recurrent laryngeal nerve was found during the operation that ascended along the medial edge of the superior thyroid pole and finally disappeared beneath the superior cornu of the thyroid cartilage without any tracheal, esophageal, or laryngeal branches. The patient was discharged on the third postoperative day with the diagnoses of papillary thyroid carcinoma and congenital left vocal cord paralysis.

**Conclusions:**

The novel variation of the recurrent laryngeal nerve may challenge the current concept of the anatomy of the nerve. The vocal folds mobility should be examined routinely before surgery in patients undergoing thyroid operation.

**Electronic supplementary material:**

The online version of this article (doi:10.1186/s12893-017-0263-5) contains supplementary material, which is available to authorized users.

## Background

Injury to the recurrent laryngeal nerve (RLN) is one of the most severe complications of thyroid surgery. Surgeons must have a comprehensive understanding of the anatomy of the RLN during thyroid operation. As direct identification and intraoperative neuro-monitoring of the RLN have become standard practices, the rate of intraoperative damage to the nerves decreased dramatically [1–3]. Nevertheless, several anatomic variations of the RLN, such as extralaryngeal branches, distorted RLN, intertwining between branches of the RLN and the inferior thyroid artery and non-recurrent laryngeal nerve, might increase the risk of iatrogenic damage [4].

## Case presentation

A 50-year-old woman was presented to our department with a thyroid nodule. She reported no dysphagia, respiratory compromise, hoarseness, chronic coughing, or loss of voice in high pitch ranges. Her medical history included breast cancer, and she had undergone a modified radical left mastectomy. She had no history of surgery or radiation to the head or neck. The patient was a farmer. She lived in the South-Central China and had not traveled outside. She was a nonsmoker and didn’t drink alcohol.

On physical examination, the patient was afebrile. The pulse was 76 beats per minute, the blood pressure 126/75 mmHg, and the respiratory rate 20 breaths per minute. The neck was supple, and a nodule was palpable in the thyroid on the right side. There was no palpable lymphadenopathy in the cervical region. The rest of the examination was normal.

**Fig. 1:**
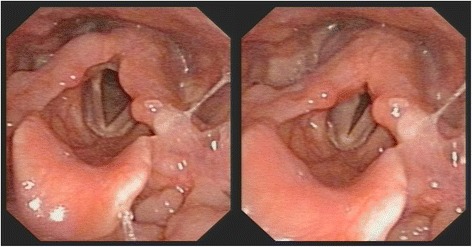
Left vocal cord paralysis diagnosed by the preoperative laryngoscopy

The blood level of thyrotropin was 2.56 μIU per milliliter (reference range, 0.35 to 4.94). Ultrasonography of the thyroid gland revealed a solid and hypoechoic nodule measuring 18 × 16 mm, including cystic necrotic areas in the mid pole of the right thyroid lobe. The nodule had lobulated margins, scattered central calcification, and rich central blood flow. There was no enlarged lymph node in the cervical region. The result of fine-needle aspiration cytology showed papillary carcinoma. The preoperative laryngoscopy revealed left vocal cord paralysis (Fig. 1). Right thyroid lobectomy was scheduled for the patient.

**Fig. 2:**
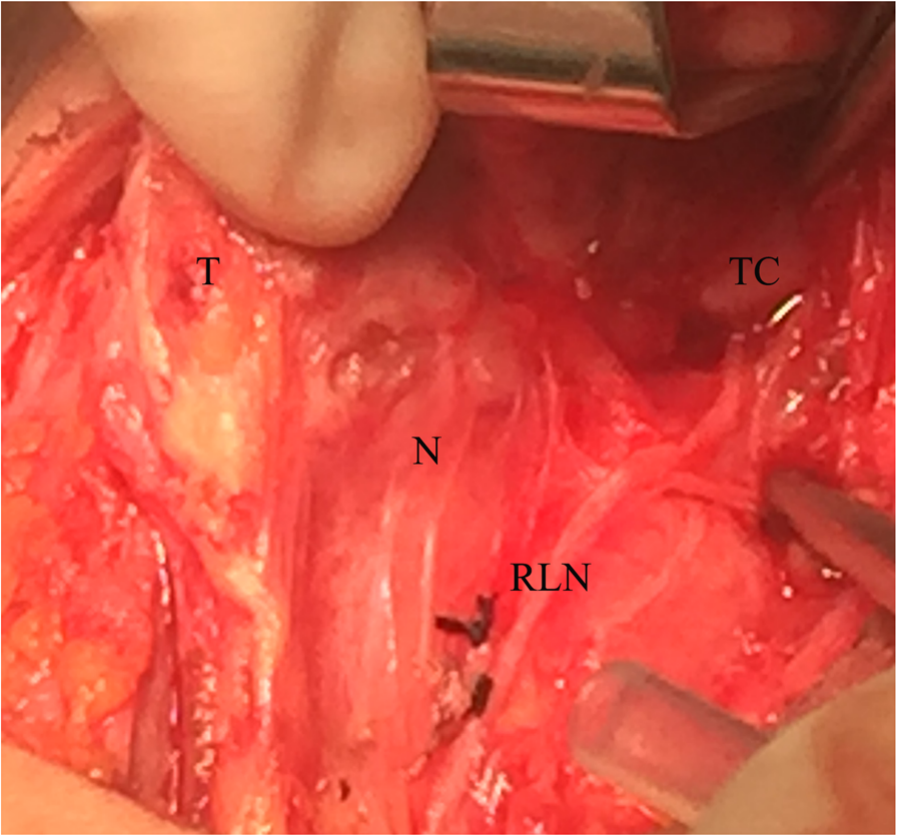
Photo of the new variation of recurrent laryngeal nerve. (T: thyroid gland; N: normal entry; RLN: recurrent laryngeal nerve; TC: thyroid cartilage)



Thyroid lobectomy with Wu Gaosong’s procedure was performed and the nerve monitoring (NIM-Response 3.0 System, Medtronic) was applied during the operation [5, 6]. We resected the thyroid gland from the inferior pole to the superior pole to allow for a good visualization of the RLN, sternothyroid–laryngeal triangle and the external branch of the superior laryngeal nerve (EBSLN). The right RLN and EBSLN were identified and found to follow the usual course of the nerve. An electromyography activity with amplitude of 724 uV was recorded when the right RLN was stimulated by the nerve stimulator. After freeing and medially mobilizing the left thyroid gland, an exploration was made for the left RLN. The left RLN was identified below the inferior thyroid artery. When the left RLN was dissected superiorly toward the cricothyroid muscle and larynx, it was seen to ascend along the medial edge of the superior thyroid pole and finally disappear beneath the superior cornu of the thyroid cartilage without any tracheal, esophageal, or laryngeal branches (Fig. 2, Additional file 1). On electromyography monitorization, no electromyography activity was recorded when the left RLN and vagus nerve were stimulated. All the nerves were carefully preserved.

**Additional file 1:** The novel variation of left recurrent laryngeal nerve. The left recurrent laryngeal nerve ascends along the medial edge of the superior thyroid pole and finally disappears beneath the superior cornu of the thyroid cartilage without any branches. (MP4 61074 kb)


Postoperatively, the patient’s voice and serum calcium were normal. Left vocal cord paralysis was reported on the postoperative laryngoscopy of the patient. The final pathology was papillary thyroid carcinoma. The patient was discharged on the third postoperative day with the diagnoses of papillary thyroid carcinoma and congenital left vocal cord paralysis.

## Discussion and conclusions

The RLN was the principal nerve which controlled vocal fold mobility through laryngeal branches. Injury to the RLN can result in voice impairments ranging from a weakened hoarse voice in unilateral lesions to respiratory distress in bilateral damage. In severe cases, urgent tracheotomy is needed as a life-saving intervention. Injury to the RLN after thyroid operations is also one of the reasons for medico-legal litigation. The incidences of permanent and transient injury of RLN after surgery were 0.3–3% and 3–8%, respectively [7]. Modern surgical strategy for preserving the integrity of the RLN included intraoperative neural monitoring and direct visualization of the nerve. Some rare anatomic variations still threatened the RLN during thyroid surgery, even if the surgeon is completely familiar with thyroid and RLN anatomy.

As far as we know, the innovative variation of RLN in this case has not previously been reported. The merit of the new variation found in this study is that the nerve ascends directly to the superior cornu of the thyroid cartilage without any tracheal, esophageal, or laryngeal branches. Full tracking of the course of this nerve was not achieved on account of the exposure limit of the incision. Notwithstanding, we hypothesize that the nerve may not enter the larynx to dominate the intrinsic muscles within the larynx considering the preoperative paralysis of the left vocal cord and intraoperative electromyography inactivity. The new variation of RLN is a significant finding for clinical practice, and the function of the nerve requires further studies.

The new variation of RLN may threaten the nerve during operation, principally in two aspects: first, ipsilateral vocal cord paralysis negates the effects of the intraoperative neuro-monitoring; second, the portion of the nerve that travels between the superior thyroid pole and the cricothyroid muscle is in danger of involuntary damage because surgeons often ligate the superior thyroid vessels at their penetration point of the thyroid for the safety of the EBSLN [8]. Moreover, the vocal fold mobility of the patients should be examined preoperatively even though the patient’s voice is normal.

Congenital causes of vocal fold paralysis include tracheoesophageal fistula, vascular ring, dysmorphic syndromes (Mobius, Goldenhaar), syndromes involving brainstem dysfunction, and neuromuscular disorders such as Charcot–Marie–Tooth [9]. The paralysis caused by inborn anomalies of the RLN has not previously been reported. More studies are needed to confirm this brave conjecture.

Our study also has some limitations. Some large sympathetic branches could be misinterpreted as RLN, especially if it is impossible to rely on intraoperative nerve monitoring. Besides, there might be some tiny branches that have been far too small to be visualized considering the potential role of anastomoses between laryngeal nerves [10].

In conclusion, we have described a novel variation of the RLN which may challenge the contemporary concept of the anatomy of the nerve. The vocal folds mobility should be examined routinely before surgery in patients undergoing thyroid operation.

## Abbreviations

EBSLN: External branch of the superior laryngeal nerve
RLN: Recurrent laryngeal nerve

## References

[1] Hermann M, Alk G, Roka R, Glaser K, Freissmuth M (2002). Laryngeal recurrent nerve injury in surgery for benign thyroid diseases: effect of nerve dissection and impact of individual surgeon in more than 27,000 nerves at risk. Ann Surg.

[2] Chandrasekhar SS, Randolph GW, Seidman MD, Rosenfeld RM, Angelos P, Barkmeier-Kraemer J, et al. Clinical practice guideline: improving voice outcomes after thyroid surgery. Otolaryngol Head Neck Surg. 2013;148:S1–S37. 10.1177/0194599813487301 23733893

[3] Randolph GW, Dralle H, International Intraoperative Monitoring Study G, Abdullah H, Barczynski M, Bellantone R, et al. Electrophysiologic recurrent laryngeal nerve monitoring during thyroid and parathyroid surgery: international standards guideline statement. Laryngoscope. 2011;121(Suppl 1):S1–16. 10.1002/lary.21119 21181860

[4] Chiang FY, Lu IC, Chen HC, Chen HY, Tsai CJ, Hsiao PJ, et al. Anatomical variations of recurrent laryngeal nerve during thyroid surgery: how to identify and handle the variations with intraoperative neuromonitoring. Kaohsiung J med Sci. 2010;26(11):575–83. 10.1016/S1607-551X(10)70089-9 PMC11916884 21126710

[5] Wu G, Kong D. Thyroidectomy with Wu Gaosong's procedure. Video Endocrinology. 2016; doi:10.1089/ve.2015.0050.

[6] Wu G, Wang K. Intraoperative Neuromonitoring and protection of the superior laryngeal nerve with Wu Gaosong's procedure. Video Endocrinology. 2016; doi:10.1089/ve.2016.0070.

[7] Hayward NJ, Grodski S, Yeung M, Johnson WR, Serpell J (2013). Recurrent laryngeal nerve injury in thyroid surgery: a review. ANZ J Surg.

[8] Barczynski M, Randolph GW, Cernea CR, Dralle H, Dionigi G, Alesina PF, et al. External branch of the superior laryngeal nerve monitoring during thyroid and parathyroid surgery: international neural monitoring study group standards guideline statement. Laryngoscope. 2013;123:S1–S14. 10.1002/lary.24301 23832799

[9] Syamal MN, Benninger MS (2016). Vocal fold paresis: a review of clinical presentation, differential diagnosis, and prognostic indicators. Curr Opin Otolaryngol Head Neck Surg.

[10] Henry BM, Pekala PA, Sanna B, Vikse J, Sanna S, Saganiak K, et al. The Anastomoses of the recurrent laryngeal nerve in the larynx: a meta-analysis and systematic review. J Voice. 2016; 10.1016/j.jvoice.2016.11.004 27939121

